# Frontoparietal network resilience is associated with protection against cognitive decline in Parkinson’s disease

**DOI:** 10.1038/s42003-021-02478-3

**Published:** 2021-09-01

**Authors:** Arianna D. Cascone, Stephanie Langella, Miriam Sklerov, Eran Dayan

**Affiliations:** 1 Neuroscience Curriculum, University of North at Chapel Hill, Chapel Hill, NC United States; 2 Department of Psychology and Neuroscience, University of North Carolina at Chapel Hill, Chapel Hill, NC United States; 3 Department of Neurology, University of North Carolina at Chapel Hill, Chapel Hill, NC United States; 4 Department of Radiology and Biomedical Research Imaging Center, University of North Carolina at Chapel Hill, Chapel Hill, NC United States

**Keywords:** Parkinson's disease, Network models

## Abstract

Though Parkinson’s disease is primarily defined as a movement disorder, it is also characterized by a range of non-motor symptoms, including cognitive decline. The onset and progression of cognitive decline in individuals with Parkinson’s disease is variable, and the neurobiological mechanisms that contribute to, or protect against, cognitive decline in Parkinson’s disease are poorly understood. Using resting-state functional magnetic resonance imaging data collected from individuals with Parkinson’s disease with and without cognitive decline, we examined the relationship between topological brain-network resilience and cognition in Parkinson’s disease. By leveraging network attack analyses, we demonstrate that relative to individuals with Parkinson’s disease experiencing cognitive decline, the frontoparietal network in cognitively stable individuals with Parkinson’s disease is significantly more resilient to network perturbation. Our findings suggest that the topological robustness of the frontoparietal network is associated with the absence of cognitive decline in individuals with Parkinson’s disease.

## Abstract (web summary)

Arianna Cascone et al. examine the relationship between frontoparietal brain network resilience and cognitive decline in Parkinson’s disease (PD). Their results suggest that individuals with PD and cognitive decline exhibit reduced tolerance to network "attacks" or perturbation in the frontoparietal network, compared to both individuals with PD with normal cognition and healthy, non-PD controls.

## Introduction

Parkinson’s disease (PD) is a progressive neurodegenerative disorder characterized by the cardinal motor symptoms of resting tremor, rigidity, bradykinesia, and postural instability^1,2^. However, a broad spectrum of nonmotor symptoms often accompanies PD as well^3^, among the most common of which is cognitive decline^4–6^. Critically, nonmotor symptoms, including cognitive impairment, profoundly impact quality of life in individuals with PD^7^. Notwithstanding its centrality in the clinical expression of the disease, the mechanisms that underlie cognitive decline in PD remain poorly understood.

Though the overall prevalence of cognitive decline in PD is high, its onset, progression, and severity are variable. Many individuals live with PD for years without experiencing cognitive deficits^4,8^. The cognitive deficits that occur in PD exist on a spectrum, from mild cognitive impairment (MCI) to moderate or severe dementia^5,9^. Though dementia can affect individuals with PD, cognitive outcomes are generally highly variable, and not all individuals with PD who experience MCI go on to develop dementia^9^. Moreover, some individuals with PD–MCI remain cognitively stable throughout the disease course and others may even experience improvement in their cognitive symptoms^4,10^. Importantly, there is also heterogeneity in domain-specific cognitive deficits observed in PD, as individuals with PD with and without dementia experience deficits in memory and visuospatial domains^11,12^. These deficits are thought to be related to disruption of the frontostriatal dopamine network as well as alterations in structural and functional properties of frontoparietal network regions^13–15^.

Indeed, neuroimaging studies have begun to uncover the neurobiological mechanisms that may underlie cognitive dysfunction in PD^16^. PD is characterized pathologically by degeneration of nigrostriatal dopaminergic neurons^17^, which provide substantial dopaminergic input to the globus pallidus and substantia nigra of the basal ganglia via the dorsal striatum, namely the caudate and putamen^16,18^. Critically, progressive loss of dopaminergic projections to the caudate nucleus correlates significantly with performance on assessments of cognitive function and with the degree of dementia in PD^19,20^. Moreover, dopamine-transporter availability in the caudate is associated with cognitive function in PD^21,22^. It has also been shown that during executive-function tasks, individuals with PD and cognitive impairment exhibit reduced activation of the caudate nuclei relative to individuals with PD without cognitive impairment^23^. Additionally, functional connectivity of the caudate correlates with cognition in PD, such that lower caudate connectivity is associated with worse global cognition scores^24^. This relationship between cognitive impairment and reduction of caudate function is not merely a reflection of more advanced PD correlating with worsened cognitive function^24^.

Network-level dysfunction has also been observed in individuals with PD and cognitive decline, especially among frontoparietal brain regions^14^. Individuals with PD–MCI and PD–dementia have altered metabolic network activity in frontal and parietal cortices, as well as extensive gray-matter atrophy and white-matter microstructural alterations within these areas^25–27^. Functional neuroimaging studies have also suggested that cognitive decline in individuals with PD and dementia is associated with disrupted functional connectivity among frontal and parietal brain regions^28,29^.

Though many neuroimaging studies examined the neural substrates of cognitive dysfunction in PD, a mechanistic account for the large variability in trajectories of cognitive decline among individuals with PD is missing. The examination of brain-network resilience, which quantifies the degree to which the topological integrity of a brain network is maintained following perturbation^30^, is a promising avenue of research. Human brain networks are robust and can withstand considerable perturbation, which is often simulated using targeted attacks^31,32^. A targeted-attack approach involves the systematic removal of nodes within a network. This can be based, for example, on nodal degree, which is the number of edges incident to each node in the network^33^. Following the targeted removal of highly connected nodes, the topological properties of functional networks in the healthy brain are largely retained^34^. The attack tolerance of brain networks in PD specifically has yet to be examined. Therefore, we reasoned that quantifying and comparing brain-network resilience across individuals with PD who experience cognitive decline and those who do not would provide insight into the mechanisms underlying the variability in cognitive dysfunction in PD.

In the current study, we thus considered the hypothesis that brain-network resilience protects against cognitive decline in PD. To test this hypothesis, we evaluated whether individuals with PD with and without longitudinal cognitive decline exhibit differing levels of topological resilience against targeted attacks. Our analysis focused on whole-brain resilience against targeted attacks, as well as the resilience of the frontoparietal network, a large-scale functional network with extensive functional connections to the caudate^35,36^. We also compared the groups across measures of whole-brain and network-level average nodal degree so as to confirm the specificity of resilience as a protective factor against cognitive decline in PD.

## Results

**Fig. 1:**
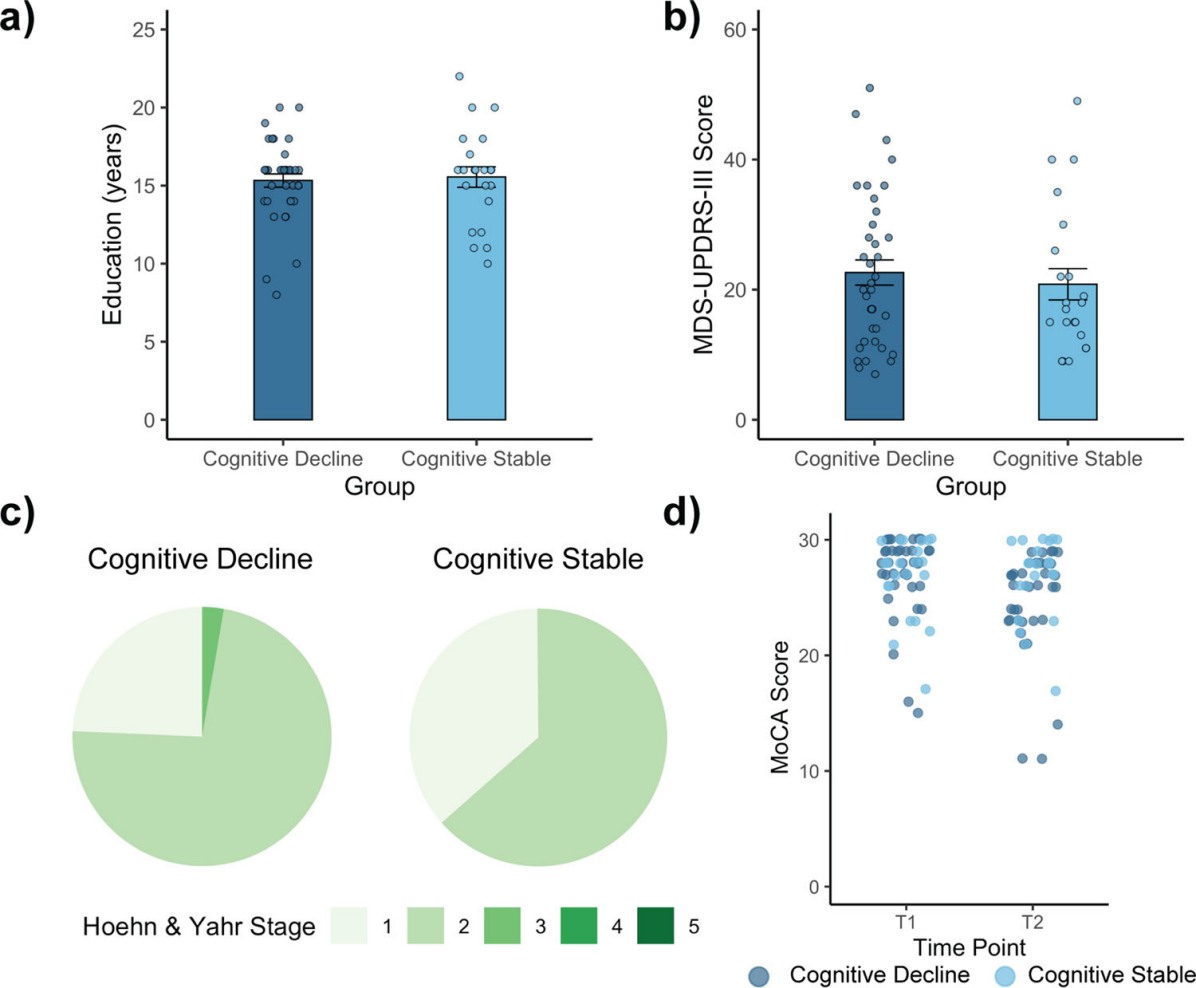
Comparison of clinical variables in subjects with Parkinson’s disease (PD). **a** Cognitively declining and cognitively stable individuals with PD did not differ in their education levels (independent-sample *t*-test, *p* = 0.77). **b** Cognitively declining and cognitively stable individuals with PD did not differ on Movement Disorder-Society—Unified Parkinson’s Disease Rating Scale-III (MDS-UPRS-III) ratings (independent-sample *t*-test, *p* = 0.57). **c** Relative distributions of Hoehn and Yahr stages for each clinical group did not differ (chi-squared test, *p* = 0.48). **d** Patients in the cognitively declining group experienced a decrease in their Montreal Cognitive Assessment (MoCA) score across time points examined, whereas patients in the cognitively stable group did not experience changes in MoCA scores. Note that the data were jittered for visualization purposes. Comparisons reported here are between *n* = 37 cognitively declining individuals and *n* = 22 cognitively stable individuals. Error bars in panels A and B depict SEM.

We analyzed neuroimaging and phenotypic data from two groups of individuals with PD: cognitively declining and cognitively stable (See “Methods”; Table 1). The PD groups did not differ in age (*t*_57_ = 0.348, *p* = 0.73), male/female distribution (*χ*^2^(1) = 1.95, *p* = 0.16), or education (*t*_57_ = −0.296, *p* = 0.77) (Fig. 1a). Moreover, the PD groups showed no differences in major clinical variables including motor function, as assessed with the Movement Disorder Society-Unified Parkinson’s Disease Rating Scale-III (MDS-UPDRS-III; *off* drug scores, only; *t*_57_ = 0.577, *p* = 0.57) (Fig. 1b), affected-side distribution (*χ*^2^(2) = 3.41, *p* = 0.18), or Hoehn and Yahr stage distribution (*χ*^2^(2) = 1.46, *p* = 0.48) (Fig. 1c). The PD groups did not differ in initial Montreal Cognitive Assessment (MoCA) scores (*t*_57_ = 0.2858, *p* = 0.78); however, there were indeed longitudinal differences in cognition between groups, indicated by a significant interaction between clinical group and time of evaluation (F(1,57) = 27.93, *p* < 0.0001; Supplementary Table 1) found in repeated-measures ANOVA. Importantly, there were no significant differences between patient groups in the proportion of individuals that were taking PD medication and those that were drug naive at the time of MoCA and UPDRS scoring (*χ*^2^(1) = 0.197, *p* = 0.66). We also found that there were no significant differences between patient groups in the proportion of individuals who were taking levodopa, dopamine agonist, or a combination of both at the time of MoCA_initial_ scoring (*χ*^2^(2) = 1.38, *p* = 0.50), nor at the time of UPDRS scoring (*χ*^2^(2) = 1.23, *p* = 0.54). No differences were observed in the number of months between MoCA evaluations (range_decline_ = 12–77 months, range_stable_ = 21–84 months; *t*_57_ = 0.023, *p* = 0.98), between imaging data acquisition and initial MoCA evaluation (*t*_57_ = 0.502, *p* = 0.62), between PD-symptom onset and initial MoCA evaluation (*t*_57_ = 0.308, *p* = 0.76), and between imaging data acquisition and the approximate date of PD-symptom onset (*t*_57_ = 0.252, *p* = 0.80) or formal PD diagnosis (*t*_57_ = 0.235, *p* = 0.81). As expected, however, the cognitively declining group showed a larger decrease in MoCA score over the time period examined as compared with the cognitively stable group (*t*_57_ = −5.285, *p* < 0.05) (Fig. 1d).

We also examined which specific cognitive domains were more prone to show a decline in the MoCA test within the cognitively declining PD group and found that individuals exhibited the largest percent decrease in the memory domain, followed by the language domain, and finally the attention, concentration, and working memory domain (Supplementary Table 2).

### Whole-brain targeted-attack analyses

**Fig. 2:**
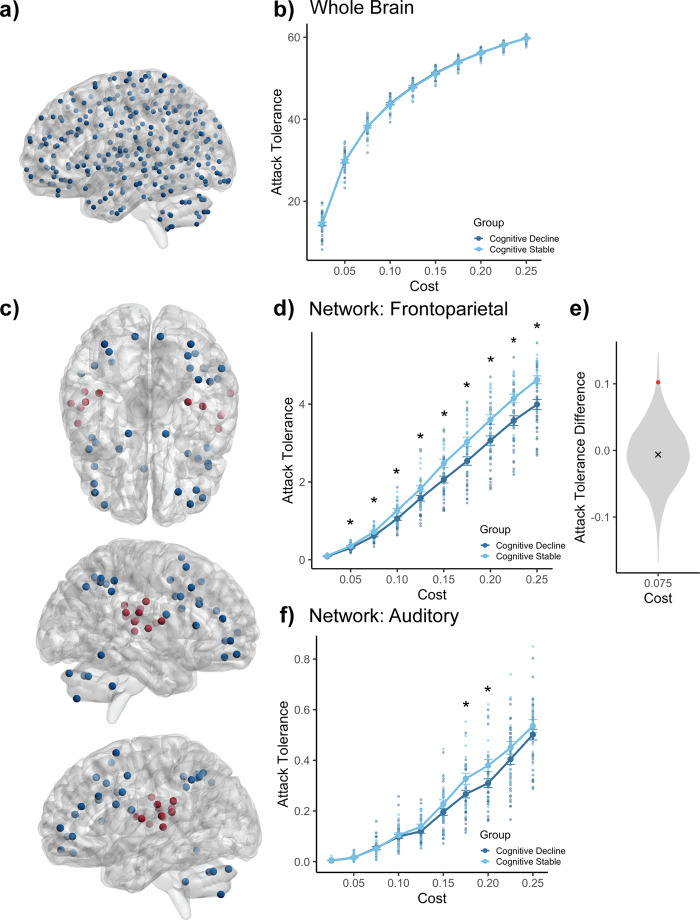
Targeted-attack analyses. Attack analyses were performed on thresholded matrices. **a** A cortico-subcortical network parcellation composed of 300 nodes was used**. b** Whole-brain targeted attacks. There were no significant differences in attack tolerance between groups across all costs examined (random-permutation test, all *p*-values > 0.66). **c** Nodes included in each network examined. Blue and red nodes correspond to the frontoparietal network and the auditory network, respectively. **d** Frontoparietal network targeted attacks. The cognitive-decline group displayed significantly reduced attack tolerance relative to the cognitively stable group across nine of the 10 costs examined (random-permutation test, *p*-value = 0.07 for cost 2.5%, otherwise all *p*-values < 0.028). **e** Distribution of *cognitively stable–cognitive decline* attack-tolerance differences at one representative cost obtained from randomized null graphs of the same size as the frontoparietal network. Hashmark denotes the average difference from this distribution, red point indicates true difference (*p*-value = 0.007). **f** Auditory network targeted attacks. There were significant differences between groups in only two of the costs examined (random-permutation test, *p*-values < 0.02 for costs 17.5 and 20%, otherwise, all *p*-values > 0.13). See Supplementary Tables 3, 4, 6, and 8 for complete summaries of statistics. Comparisons reported here are between *n* = 37 cognitively declining individuals and *n* = 22 cognitively stable individuals. Error bars depict SEM and asterisks indicate significance, *p* < 0.05.

We first examined whole-brain resilience against targeted attacks across the clinical groups, iteratively attacking the entire functional connectome, as represented in the 300-node parcellation used in the current study (Fig. 2a). Attack-tolerance values, quantified for each patient, were compared using nonparametric tests at each network cost examined (2.5–25%; Fig. 2b). No group differences were observed across all costs examined (all *p*-values > 0.66, Supplementary Table 3). While our entire cognitive decline group (*n* = 37) was composed of 17 individuals with a single-point decline in MoCA score, we confirmed that there were no significant differences in whole-brain (all *p*-values > 0.17) nor frontoparietal network (all *p*-values > 0.06) attack tolerance between subjects with a one-point change compared to those with a greater-than-one-point change in MoCA score at each of the ten costs examined.

### Single-network targeted-attack analyses

We then examined network-level resilience against targeted attacks across the clinical groups. Our analysis focused specifically on the frontoparietal network (Fig. 2c), given its extensive connectivity with the caudate, a major site of pathology in PD^16,18^, with a documented role in cognitive function^19,20^. Clinical groups differed in frontoparietal attack tolerance across nine of the 10 costs examined (all *p*-values < 0.028). More specifically, the cognitively declining group displayed significantly reduced attack tolerance relative to the cognitively stable group across all network costs, except one, 2.5% (Fig. 2d; Supplementary Table 4). We also compared attack tolerance in a subset of the cognitive-decline group who exhibited clinically relevant changes in cognitive performance (*n* = 12). To define this group, we characterized each subject in the cognitive-decline group as showing cognitively intact (CI) performance, mild cognitive impairment (PD–MCI), or dementia (PDD) at the initial and final MoCA evaluations^37^. We found similar differences in attack tolerance when comparing the subset of individuals who experienced cognitive decline and a change in their cognitive status to the cognitively stable group (all *p*-values < 0.036; Supplementary Table 5). We further assessed the significance of this finding by performing a random-permutation test (*n* = 5000 iterations) where attack tolerance in both clinical groups was compared in random networks of the same size as the frontoparietal network (36 nodes) across the different costs considered in previous analyses (Fig. 2e, Supplementary Table 6). We determined that the real difference in attack tolerance across groups was consistently higher than that of the random-difference scores across all costs examined (all *p*-values < 0.01; Supplementary Table 6). Additionally, we found that attack tolerance in the frontoparietal network was associated with changes in MoCA score in the cognitively declining group across six of the 10 costs we examined (all *p*-values < 0.05), but that there were no relationships between attack tolerance in the frontoparietal network and motor-symptom severity (all *p*-values > 0.456), nor between decline in MoCA score and MDS-UPDRS-III score (*p* = 0.649) in this group.

We also examined attack tolerance of the frontoparietal network after excluding cerebellar nodes within the network. There were similar patterns in attack tolerance across the two groups in the analyses that included (Fig. 2d) and excluded (Supplementary Fig. 1) cerebellar nodes, in that the cognitively declining group displayed reduced attack tolerance relative to the cognitively stable group across costs. There were significant differences across four of the 10 costs examined (all *p*-values < 0.047; Supplementary Table 7).

We next examined whether the differences in attack tolerance reported above were specific to the frontoparietal network, or rather were observed in other single functional networks. As a control network, we examined the auditory network, a large-scale functional network composed of regions in the primary auditory cortex and peripheral auditory regions in the superior temporal gyrus (Fig. 2c)^38^. This network was chosen as a control network as it has no major involvement in PD. Indeed, in eight out of 10 costs considered, there were no significant group differences in attack tolerance (Fig. 2f and Supplementary Table 8).

### Specificity of resilience to targeted attack as a topological property

**Fig. 3:**
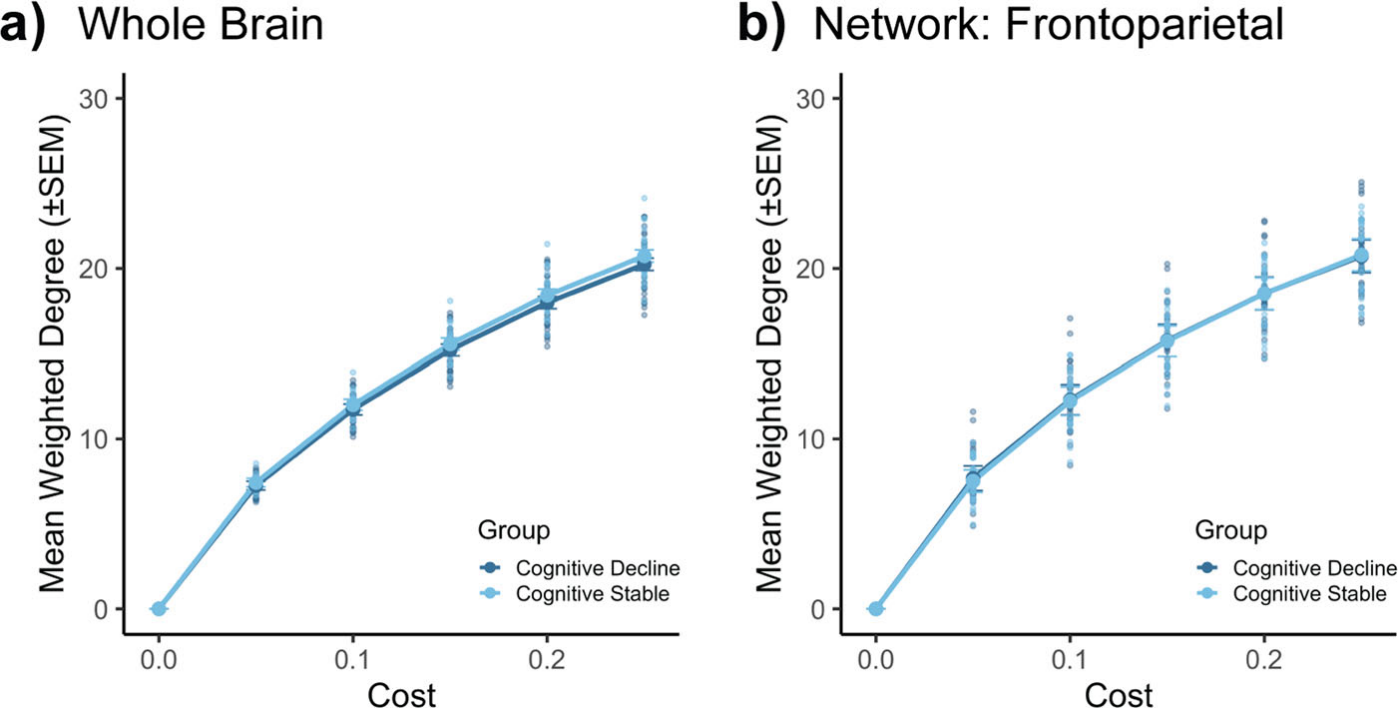
Weighted-degree analyses. Mean (±SEM) weighted degree (‘strength’) of (**a**) all 300 nodes and of (**b**) nodes within the frontoparietal network, specifically. Weighted degree (**a, b**) was derived from thresholded matrices. There were no significant differences across groups (all *p*-values > 0.21). Comparisons reported here are between *n* = 37 cognitively declining individuals and *n* = 22 cognitively stable individuals. See Supplementary Tables 9 and 10 for complete summaries of statistics.

Given that the PD groups showed differences in attack tolerance in the frontoparietal network, we considered the option that these differences reflect general topological differences between these two groups. Thus, we went on to examine whether there were differences in whole-brain and frontoparietal network weighted degree (also referred to as “strength”; Fig. 3; Supplementary Tables 9–10). The two PD groups did not differ in whole-brain (Fig. 3a) nor single-network measures (Fig. 3b) of weighted degree across any of the costs examined (all *p*-values > 0.21).

We also examined the overall functional connectivity of the frontoparietal network in both clinical groups in an effort to confirm that resilience to targeted attacks of the network was not the result of simple functional connectivity differences or confounds originating from proportional thresholding of connectivity matrices^39^. There were no significant differences in functional connectivity of the frontoparietal network across clinical groups when positive correlations were retained (*t*_57_ = 1.32, *p* = 0.19), nor when examining the absolute values of all correlations (*t*_57_ = 1.00, *p* = 0.32).

### Examining frontoparietal network attack and weighted-degree analyses in healthy controls

**Fig. 4:**
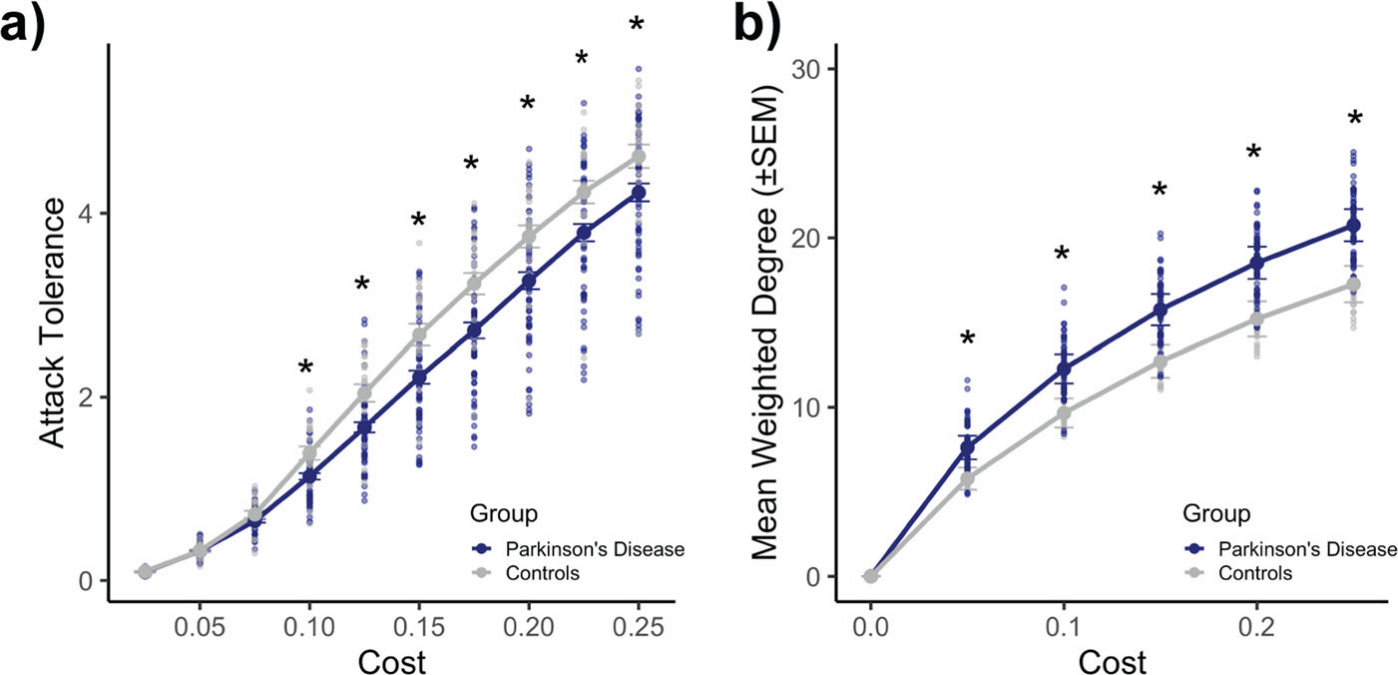
Parkinson’s disease vs. control group comparisons of attack tolerance and weighted degree of the frontoparietal network. All analyses were performed on thresholded matrices. **a** Targeted-attack analysis. The PD group displayed significantly reduced attack tolerance relative to the healthy control group across seven of the 10 costs examined (random-permutation test, *p*-values > 0.08 for costs 2.5–7.5%; otherwise, *p*-values < 0.03). **b** Mean (±SEM) weighted degree of nodes within the frontoparietal network. The control group displayed significantly reduced weighted degree across all of the valid costs examined (random-permutation test, all *p*-values < 0.0002). Comparisons reported here are between *n* = 59 individuals with PD and *n* = 21 controls. See Supplementary Tables 11 and 13 for complete summaries of statistics.

We next wished to test whether attack-tolerance differences in the frontoparietal network were specific to cognitive decline in PD, or rather reflected more general differences observed between patients and controls. We first ensured that there were no significant differences in age (*t*_78_ = −0.185, *p* = 0.85), male/female distribution (*χ*^2^(1) = 1.63, *p* = 0.20), or initial MoCA score (*t*_78_ = −1.088, *p* = 0.28) between patients and controls. We examined frontoparietal resilience against targeted attacks across the clinical and control groups (Fig. 4a). Combining the two clinical groups, we found that PD patients displayed significantly reduced attack tolerance relative to controls at all but three network costs (*p*-values < 0.03) (Supplementary Table 11). Comparing the two PD groups with the control groups separately, we found that the differences in attack tolerance between the PD and control groups were specific to the cognitively declining PD group. That is, the cognitively declining PD group displayed significantly reduced attack tolerance relative to the control group (all adjusted *p*-values < 0.03), but there were no differences in attack tolerance between the cognitively stable PD group and controls (all adjusted *p*-values > 0.08; Supplementary Table 12). It is likely that the attack-tolerance differences between the PD and control groups are reflective of basic network topology, as the control group displayed significantly reduced weighted degree in the frontoparietal network relative to patients at all valid costs examined (all *p*-values < 0.0002) (Fig. 4b; Supplementary Tables 13–14).

## Discussion

Cognitive decline is one of the most common nonmotor symptoms in PD, yet the onset, progression, and severity of cognitive dysfunction are variable across individuals with the disease^4,8^. The mechanisms underlying variability in cognitive-decline trajectories remain unknown. In the current study, we examined the relationship between brain-network resilience and cognitive decline in PD by leveraging targeted-attack analyses on functional connectomes. We found that there was an association between brain-network resilience of the frontoparietal network and the absence of cognitive decline in individuals with PD. That is, relative to cognitively declining individuals with PD, cognitively stable individuals with PD displayed greater resilience to targeted attacks within the frontoparietal network. These two groups of patients did not exhibit differences in overall functional connectivity within the frontoparietal network or in more general topological properties (i.e., weighted degree) of this network. Further, we determined that the results were specific to the frontoparietal network, finding no group differences across eight of the 10 costs we examined in response to targeted attacks of a network with no major role in PD (i.e., auditory network). It is likely that the significant differences between auditory network-attack tolerance we found across groups is reflective of cost-dependent differences, rather than true group differences, as there were only significant differences in two of the 10 costs examined^40^.

We evaluated brain-network resilience in PD by quantifying the tolerance exhibited by individual subjects’ brain networks to targeted attacks. We report that cognitively stable individuals with PD exhibit stronger tolerance to targeted attacks in the frontoparietal network than individuals with PD and declining cognition. The resilience of networks to insult and perturbation has been widely examined in the context of biological, social, and artificial systems^33,41–43^. The robust organization of brain networks renders them resilient, such that the topological integrity of brain networks is highly conserved in the face of degeneration or disease^30^. In the context of our findings, this could mean that the brain networks of some individuals with PD can withstand considerable damage while maintaining key features of topology that are critical for normal cognition. The ability of brain networks to withstand perturbation may also originate from the existence of redundancy in cortico-subcortical brain networks^44,45^, which may differ among individual patients. That is, the brain networks of individuals with PD may vary in their ability to sustain the same amount of perturbation, which would become evident following the progression of neuropathology.

Our findings document differences in brain-network resilience in the frontoparietal network, a large-scale functional network involved in higher-order cognitive function, with intricate functional connections to the caudate^46^. We did not find group differences in whole-brain-network resilience, in the resilience of random networks of the same size as the frontoparietal network, or in resilience of the auditory network^47,48^, analyzed here as a control network, as it is not typically associated with cognition, nor substantially implicated in PD. Our findings thus reveal specificity with respect to the resilience of the frontoparietal network. These results fit in with previous work that has revealed the central role of the caudate nucleus and the frontoparietal network in cognition in PD^19,20,23^. In particular, aberrant connectivity in the caudate and the frontoparietal network relates to cognitive function in PD^24,49^. Our results therefore add evidence to corroborate the caudate and frontoparietal network’s critical roles in cognition and in the etiology of cognitive decline in PD.

In our primary analysis, we examined resilience of the entire frontoparietal network, including cerebellar nodes, as included in the cortico-subcortical parcellation used in this study^35^. There is evidence to suggest that cerebellar regions are integrated with large-scale functional networks, and that cerebellar nodes affiliate with well-characterized networks, including the frontoparietal network^35,50,51^. Since previous functional neuroimaging work has found that cerebellar regions coactivate with the frontoparietal cortices during cognitive tasks^52^ and that cerebellar regions receive input from prefrontal and parietal regions^53,54^, we wished to further examine the role of cerebellar nodes in attack tolerance. Our results highlight a central role for cerebellar nodes of the frontoparietal network in brain-network resilience. These findings fit in well with previous studies that have examined functional activation and connectivity in PD and have found hyperactivation in cerebellar regions during tasks with substantial cognitive demands^55^. Increased cerebellar activity has also been related to better cognitive performance in individuals with PD^56^. This hyperactivation is thought to represent a compensatory function that may mitigate the early symptoms of neurodegeneration in PD^57,58^. That is, the cerebellum may be maintaining brain-network resilience when other parts of the brain (i.e., dopaminergic basal ganglia regions) are challenged with PD pathology. The present results add to the current literature and provide support for these contentions, as our results suggest that the resilience of the frontoparietal network, particularly with cerebellar ROIs included, is associated with the absence of cognitive decline in this population.

To assess whether the findings on frontoparietal network resilience in PD were reflective of, or driven by, more general topological differences, we tested if the two clinical groups also differed on a basic measure of network topology, weighted degree (“strength”), or on the overall functional connectivity of the frontoparietal network. This is noteworthy because metrics derived from functional connectivity matrices are influenced by basic topological characteristics^30^. We observed no differences in weighted degree nor in the overall functional connectivity of the frontoparietal network. These auxiliary findings therefore suggest that our analytic method taps a distinct feature of network organization in PD. Put differently, our findings suggest that the differences in cognition evident in PD are more strongly driven by differences in topological robustness, rather than by more basic topological network attributes or simple functional connectivity differences^24^. It was previously also found that after a prolonged period of disease (i.e., eight years), hyperconnectivity in frontal brain regions, revealed via analysis of electroencephalogram (EEG) data, can be a predictor of cognitive decline in PD^59^. However, methodological differences in comparison with the present study (including the use of different imaging modalities and large difference in disease duration) may preclude direct comparisons. Future studies may examine the topology of functional connectomes in PD using other imaging modalities and clinical samples with a larger range of disease duration.

We also compared frontoparietal network resilience to targeted attacks across patient and control groups. We found that healthy controls were significantly more resilient to targeted attacks, but that there were basic topological differences (i.e., in weighted degree) between patient and control groups in this network. Given this finding, and that network measures can indeed be influenced by basic features of topology^30^, these results were likely reflective of general topological differences between the clinical and control groups. These results are in line with previous work, which has shown marked differences in functional network topology between individuals with PD and age-matched controls^13,60,61^. More specifically, similar to the results reported here, previous studies have also found significantly reduced degree in the functional connectomes of age-matched controls relative to those of individuals with PD^60,61^.

Several limitations should be discussed. Our findings suggest that the topological robustness of the frontoparietal network may be protective against cognitive decline in PD, though we used cross-sectional imaging data along with longitudinal measures of cognition. Future studies may use longitudinal imaging data to further corroborate our findings. Additionally, we used a global measure of cognition, the MoCA, to assess longitudinal changes in cognition. The MoCA has been used successfully as a valid and reliable^62^ screening tool for cognitive impairment in PD^63,64^; however, future studies may examine cognition longitudinally using more specific measures.

In conclusion, we found that relative to individuals with PD experiencing cognitive decline and age-matched controls, the frontoparietal network in cognitively stable individuals with PD is significantly more resilient to targeted attacks. Our findings suggest that the topological robustness of the frontoparietal network is associated with the absence of cognitive decline in individuals with PD.

## Methods

### Subjects

All data in the present study were extracted from the Parkinson’s Progression Markers Initiative (PPMI) database (http://www.ppmi-info.org). PPMI is a comprehensive observational, international, multicenter study designed to identify progression markers of PD^65^. All participants provided written informed consent, and the procedures were all in accordance with the approved regulations and guidelines of the Institutional Review Boards of participating study centers.

Participants with PD for the present study were initially chosen based on the presence of complete anatomical and resting-state functional MRI (fMRI) data and longitudinal evaluation of global cognition at the time data were acquired from the PPMI database (August 2019; *n* = 101 participants). Each subject’s global cognition, as measured by scores on the MoCA (range: 0–30), at the most recent evaluation, was compared to cognition at their initial assessment (MoCA_last_ − MoCA_initial_). Individuals who experienced improvements in cognition (i.e., MoCA_last_ − MoCA_initial_ > 0) were excluded from the current analyses. Individuals who met inclusion criteria were further classified into one of two PD groups based on the presence or absence of cognitive decline. Individuals in the *cognitive decline* group were those who experienced a decline in their MoCA score of at least one point (i.e., MoCA_last_ − MoCA_initial_ < 0), whereas individuals in the *cognitively stable* group experienced no changes in MoCA scores (i.e., MoCA_last_ − MoCA_initial_ = 0). Thus, overall, data from 59 individuals with PD were analyzed, including 37 individuals who experienced cognitive decline (mean age = 62.97 ± 9.8y) and 22 individuals who were cognitively stable (mean age = 62.05 ± 10.0y). A similar proportion of subjects within these two groups (*χ*^2^(1) = 0.154, *p* = 0.69) had MoCA_initial_ scores indicative of cognitive impairment (i.e., <26). Per PPMI study cohort guidelines, the subjects with PD included in this study were medication-naive individuals at the time of enrollment with PD diagnoses of two years or less. However, most PPMI participants started taking dopaminergic medications within the first two years of the study, which is when the resting-state fMRI protocol was added. We found that of those who were taking PD medication and whose medication status at the time of scan was known, the proportion of individuals who were on/off PD medication at the time of the resting-state fMRI scan was similar across groups (*χ*^2^(1) = 0.505, *p* = 0.48). When also considering those who were drug naive in the *off*-medication group, we again did not find significant differences in the proportion of individuals who were on/off PD medication at the time of the fMRI scan between groups (*χ*^2^(1) = 0.0002, *p* = 0.99). Complete inclusion and exclusion criteria for PPMI clinical groups are available online (http://www.ppmi-info.org).

We also included age-matched *healthy controls* in the current study. Of the controls that were enrolled in the PPMI study, subjects for the current study were chosen based on the presence of complete anatomical and resting-state fMRI data at the time of data download (*n* = 22). One control subject was excluded from the analyses because of excessive head motion during scanning (see below). We did not examine cognition longitudinally in the control group, because our small sample size for controls (*n* = 21, mean age = 63.10 ± 10.3y) was underpowered to detect group differences in longitudinal cognitive outcomes (i.e., of cognitive-decline and cognitively stable subgroups).

### Clinical and cognitive evaluations

Per PPMI protocol, all subjects underwent clinical and cognitive assessments. Clinical characteristics were rated using the MDS-UPDRS, with scores from Part III of the scale (range: 0–132) used to assess motor dysfunction. PD symptom progression was also rated according to the Hoehn and Yahr system (stages 0–5). Global cognition was evaluated with the MoCA (range: 0–30) (Table 1)^63^, a cognitive screening test used to detect mild cognitive impairment.

**Table 1:** Demographic and clinical characteristics of Parkinson’s disease (PD) and control groups.

	PD groups		Control group
	Cognitive decline	Cognitive stable	
Age (years)	62.97 ± 9.84	62.05 ± 10.03	63.10 ± 10.26
Sex	22M/15F	17M/5F	17M/4F
Initial Montreal Cognitive Assessment (MoCA) score	27.00 ± 3.57	26.73 ± 3.49	27.76 ± 1.48
Final Montreal Cognitive Assessment (MoCA) score	24.68 ± 4.51	26.73 ± 3.49	
∆MoCA score	−2.32 ± 2.06	0.00 ± 0.00	
Months between MoCA evaluations	57.65 ± 15.14	57.55 ± 18.94	
Months between scan & MoCA_initial_	0.541 ± 1.99	0.318 ± 0.72	
Months between PD symptom onset & MoCAinitial	41.41 ± 40.26	38.41 ± 27.84	
Months between scan & PD symptom onset	41.14 ±4 0.10	38.68 ± 28.02	
Months between scan & formal PD diagnosis	19.24 ± 34.50	17.23 ± 26.61	
Movement Disorder Society-Unified Parkinson’s Disease Rating Scale (MDS-UPDRS)-III	22.62 ± 11.78	20.82 ± 11.31	
Affected side (left/right/asymmetric)	16/20/1	5/17/0	
Hoehn & Yahr stage (1/2/3/4/5)	9/27/1/0/0	8/14/0/0/0	
Education (years)	15.32 ± 2.59	15.55 ± 3.07	

### Imaging data acquisition

All MRI data were acquired with Siemens 3T scanners (TrioTim or Prisma). Anatomical scans were acquired using a magnetization prepared rapid acquisition gradient echo (MPRAGE) sequence with generalized autocalibrating partial parallel acquisition (GRAPPA) (TE = 2.98 ms, TR = 2300 ms, flip angle = 9˚, voxel size = 1 mm^3^). Resting-state fMRI scans were acquired using an echo-planar sequence (TE = 25 ms, TR = 2400 ms, 210 volumes, flip angle = 80˚, voxel size = 3.3 mm^3^). The resting-state scans were 8.4 min in length. During the resting-state scan, subjects were instructed to lay quietly awake in the scanner with their eyes open.

### Image preprocessing

Preprocessing of imaging data was performed via MATLAB R2019b (MathWorks, Natick, MA, USA), using SPM 12 and the CONN toolbox version 18.b^66^. Complete documentation of the following CONN pipelines can be found online (https://web.conn-toolbox.org/fmri-methods)^67^.

CONN’s default preprocessing pipeline for volume-based analyses was used. During preprocessing, functional images were realigned, unwarped, and slice-time corrected. Outlier volumes were identified and scrubbed, using the intermediate settings of 0.9-mm subject motion as measured by framewise displacement and a global signal threshold of Z = 5. Subjects (*n* = 1) with >50% of volumes removed were excluded from analyses. Functional data were normalized into standard Montreal Neurological Institute (MNI) space, and gray matter, white matter, and cerebrospinal fluid were segmented. Finally, functional data was spatially smoothed with a Gaussian kernel of 8-mm full-width half-maximum. After data preprocessing, CONN’s default denoising pipeline was used. White matter, cerebrospinal fluid, and 12 subject-motion parameters were included as regressors in the denoising step. A temporal band-pass filter was also applied to remove temporal frequencies below 0.008 Hz or above 0.09 Hz from the blood-oxygen-level-dependent (BOLD) signal.

### Matrix construction and attack analyses

**Fig. 5:**
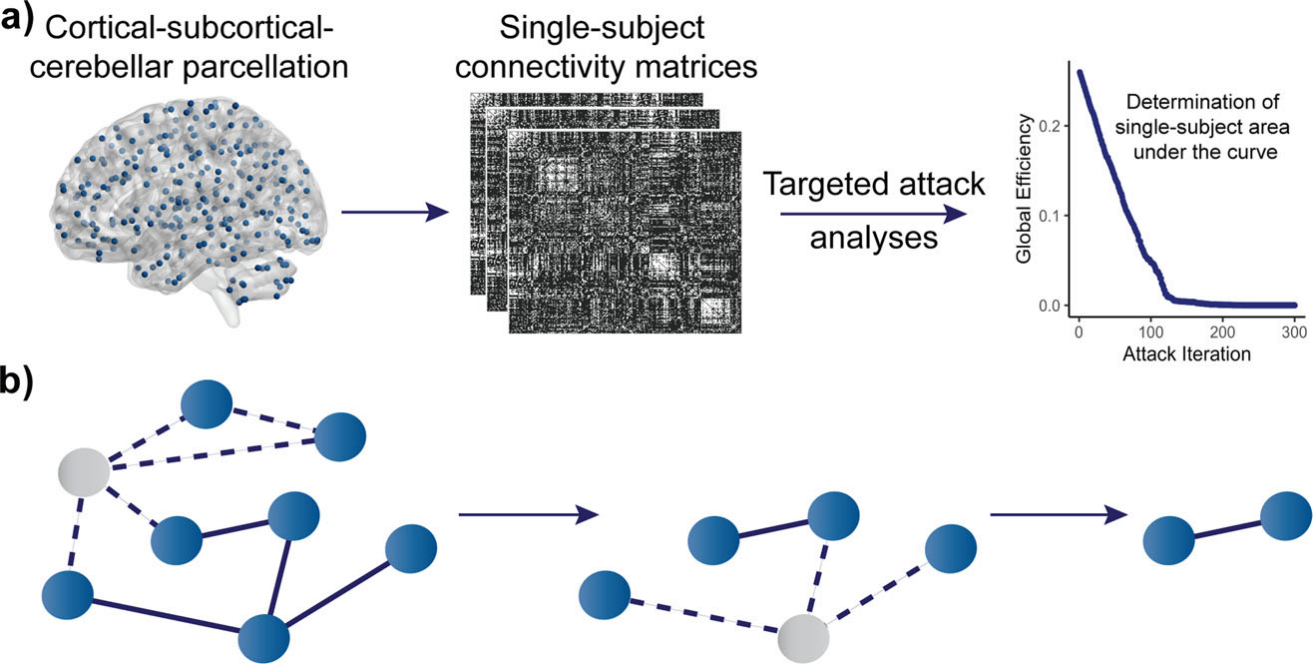
Imaging and network-attack analysis pipeline. **a** Summary of methodology. Using a functionally defined 300-node parcellation, weighted connectivity matrices were computed and binarized; network-attack analyses were performed on these single-subject connectivity matrices. **b** Network graphs depicting network nodes and edges as circles and lines. Targeted-attack analysis involves the iterative removal of nodes in an ordered fashion, starting with the node with the highest degree, followed by the node with the next-highest degree, and so on. Gray nodes and the corresponding dotted lines represent the nodes and subsequent edges removed in each attack iteration.

Functional time series were derived using a functionally defined cortical–subcortical–cerebellar parcellation of 300 nonoverlapping spherical regions of interest (ROIs)^35^. Single networks (i.e., frontoparietal and auditory networks) were defined according to the functional subdivisions outlined in Seitzman et al. (2020)^35^, where a comprehensive functionally defined cortical–subcortical parcellation was developed. Montreal Neurological Institute (MNI) coordinates for each ROI included in the frontoparietal and auditory networks can be found in Supplementary Tables 15 and 16, respectively. Unweighted connectivity matrices were computed, with the elements in each matrix expressing Fisher Z-transformed bivariate correlations between the BOLD time series for each pair of ROIs (Fig. 5a). Prior to network-attack analyses, we binarized connectivity matrices at a range of thresholds (costs) in which the top 2.5–25% of edges were preserved. We examined 10 total costs, with a 2.5% step between each.

Whole-brain and single-network targeted-attack analyses were performed using MATLAB R2019b (MathWorks, Natick, MA, USA) and the Brain Connectivity Toolbox^30^. All attack analyses were performed at all network costs (2.5–25%). Nodes were iteratively removed in descending order of degree, which is a measure of a node’s connections to other nodes (Fig. 5b)^30^. Global efficiency was calculated after each attack iteration, yielding an overall curve denoting the values of global efficiency as a function of the fraction of nodes removed/attacked. The area under the curve was then computed across all iterations and used as a measure of network resilience^68^.

#### Calculation of degree and global efficiency

*Unweighted degree* was calculated as

1 $${k}_{i}=\mathop{\sum }\limits_{j=1}^{n}{d}_{ij}$$

where *i* and *j* correspond to network nodes, and *d* is the connection between nodes^30^. We also calculated *weighted degree*, since we used proportional thresholding in our connectivity matrices. Weighted degree was calculated as

2 $${k}_{i}^{W}=\mathop{\sum }\limits_{j=1}^{n}{w}_{ij}$$

where w_ij_ describes the connection weight between nodes *i* and *j*^30^. Global efficiency, the inverse of the shortest path between two nodes^30^, was calculated as

3 $${E}_{global}=\frac{1}{n(n-1)}\,\mathop{\sum}\limits_{i,j,j\ne 1}\frac{1}{{L}_{ij}}$$

where *n* is the total number of nodes in the network, and L_*ij*_ is the shortest path length between nodes *i* and *j*.

### Statistics and reproducibility

All statistical analyses were completed in R version 4.0.2^69^. In the PD groups, demographic and scale variables were compared using either an independent-sample *t*-test or a chi-squared test. A repeated-measures analysis of variance (ANOVA) was also used to examine longitudinal changes in MoCA score between the clinical groups. In the ANOVA, time of evaluation (initial/final) and PD group (stable/decline) were included as factors. Permutation tests were used for clinical group comparisons of network resilience (AUC values, as described above) and weighted degree. Clinical group comparisons were evaluated using a nonparametric pairwise permutation test at each of the 10 costs with the aovperm function from the permuco package in R^70^. Five-thousand permutations were used for each test, and the significance level was set at *p* = 0.05.

Clinical and control groups were compared across measures of network resilience and weighted degree in the frontoparietal network, specifically. Nonparametric pairwise permutation tests were again used in the comparisons of clinical and control groups at each of the 10 costs.

### Reporting summary

Further information on research design is available in the Nature Research Reporting Summary linked to this article.

## Supplementary information

Supplementary Information

Reporting Summary

## Data Availability

Neuroimaging, cognitive and demographic data are available on the Parkinson’s Progression Markers Initiative site (https://www.ppmi-info.org/). The datasets generated in the current study are available in figshare with the identifier: 10.6084/m9.figshare.14955189^71^. All other data are available from the corresponding author upon reasonable request.
