# Supplementary material for: Frontoparietal network resilience is associated with protection against cognitive decline in Parkinson’s disease
Source: Commun Biol. 2021 Sep 1;4:1021. doi: 10.1038/s42003-021-02478-3 (PMC8410800; doi:10.1038/s42003-021-02478-3)
Supplement: Supplementary file 1 — Supplementary Information [file 42003_2021_2478_MOESM1_ESM.docx]

Supplementary Materials for:

**Frontoparietal network resilience is associated with protection against cognitive decline in Parkinson’s disease**

Arianna D. Cascone, Stephanie Langella, Miriam Sklerov and Eran Dayan


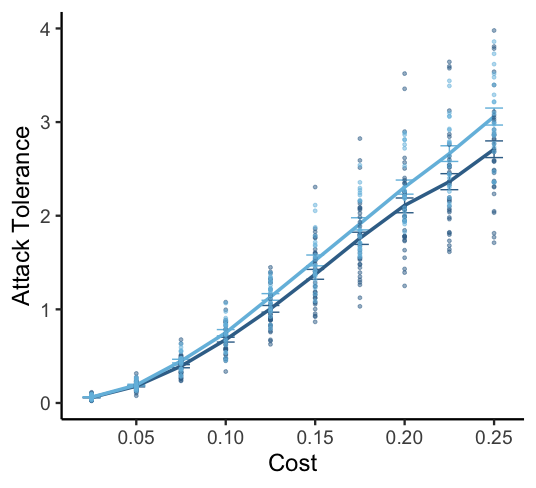


*

*

*

*


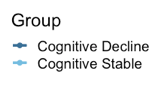


**Supplementary Figure 1. Frontoparietal network targeted attack analysis, excluding cerebellar nodes.** Attack analyses were performed on thresholded matrices. The cognitive decline group displayed significantly reduced attack tolerance relative to the cognitively stable group across four of the 10 costs examined (random permutation test, all significant p-values <0.047). See Supplementary Table 9 for complete summary of statistics. Comparisons reported here are between n=37 cognitively declining individuals and n=22 cognitively stable individuals. Error bars depict SEM and asterisks indicate significance, p<0.05.

Supplementary Table 1.

*Two-factor ANOVA results*, MoCA score over time across Parkinson’s disease groups.

|  | numDF | denDF | F-value | p-value |
| --- | --- | --- | --- | --- |
| Time of Evaluation (initial/final) | 1 | 57 | 46.9750 | <0.0001 |
| PD group (stable/decline) | 1 | 57 | 0.7639 | 0.3858 |
| Evaluation:PD Group | 1 | 57 | 27.9311 | <0.0001 |

Supplementary Table 2.

*Percent change in MoCA subscores of the cognitively declining group*.

| MoCA Domain | Percent Change |
| --- | --- |
| Memory | -13.47 |
| Language | -12.79 |
| Attention, concentration, WM | -9.46 |
| Executive | -9.01 |
| Visuospatial | -7.41 |
| Orientation | -3.60 |

Supplementary Table 3.

*Whole brain targeted attack analysis summary statistics across costs.* Values presented are mean±SEM of area under the curve.

| Cost | Parkinson’s disease groups | | P-value |
| --- | --- | --- | --- |
|  | Cognitive Decline | Cognitive Stable |  |
| 0.025 | 14.45±0.4443 | 14.55±0.3928 | 0.8704 |
| 0.050 | 29.75±0.4167 | 29.81±0.3786 | 0.9214 |
| 0.075 | 38.19±0.3214 | 38.19±0.3322 | 0.9894 |
| 0.100 | 43.81±0.2490 | 43.71±0.2685 | 0.7836 |
| 0.125 | 47.94±0.2132 | 47.83±0.2414 | 0.7544 |
| 0.150 | 51.27±0.1871 | 51.13±0.2358 | 0.6564 |
| 0.175 | 53.97±0.1615 | 53.90±0.2085 | 0.7862 |
| 0.200 | 56.23±0.1362 | 56.17±0.1782 | 0.8226 |
| 0.225 | 58.12±0.1139 | 58.10±0.1552 | 0.8978 |
| 0.250 | 59.77±0.1002 | 59.75±0.1417 | 0.9034 |

Supplementary Table 4.

*Frontoparietal network targeted attack analysis summary statistics across costs.* Values presented are mean±SEM of area under the curve.

| Cost | Parkinson’s disease groups | | P-value |
| --- | --- | --- | --- |
|  | Cognitive Decline | Cognitive Stable |  |
| 0.025 | 0.0890±0.0044 | 0.1020±0.0051 | 0.0692 |
| 0.050 | 0.3053±0.0129 | 0.3524±0.0135 | 0.0188 |
| 0.075 | 0.6127±0.0250 | 0.7149±0.0274 | 0.0112 |
| 0.100 | 1.057±0.0434 | 1.267±0.0498 | 0.0048 |
| 0.125 | 1.580±0.0707 | 1.822±0.0775 | 0.0276 |
| 0.150 | 2.062±0.0872 | 2.477±0.1072 | 0.0052 |
| 0.175 | 2.538±0.1088 | 3.031±0.1189 | 0.0048 |
| 0.200 | 3.064±0.1190 | 3.601±0.1235 | 0.0048 |
| 0.225 | 3.574±0.1249 | 4.145±0.1104 | 0.0044 |
| 0.250 | 3.989±0.1283 | 4.624±0.1010 | 0.0014 |

Supplementary Table 5.

*Frontoparietal network targeted attack analysis summary statistics across costs, cognitive decline subset vs. cognitively stable*. Here, the cognitive decline subset group was made up of PD patients who experienced a change in cognitive categorization (n=12) over the time period examined. Values presented are mean±SEM of area under the curve.

| Cost | Parkinson’s disease groups | | P-value |
| --- | --- | --- | --- |
|  | Cognitive decline subset (n=12)  Change in cognitive categorization | Cognitively stable (n=22) |  |
| 0.025 | 0.0812±0.0062 | 0.1020±0.0050 | 0.0192 |
| 0.050 | 0.3037±0.0160 | 0.3524±0.0135 | 0.036 |
| 0.075 | 0.6084±0.0338 | 0.7149±0.0274 | 0.027 |
| 0.100 | 1.056±0.0680 | 1.267±0.0498 | 0.0172 |
| 0.125 | 1.592±0.1321 | 1.822±0.0775 | 0.1132 |
| 0.150 | 2.124±0.1675 | 2.477±0.1072 | 0.0766 |
| 0.175 | 2.637±0.1809 | 3.031±0.1189 | 0.0648 |
| 0.200 | 3.206±0.1854 | 3.601±0.1235 | 0.0728 |
| 0.225 | 3.695±0.1749 | 4.145±0.1104 | 0.0336 |
| 0.250 | 4.141±0.1761 | 4.624±0.1010 | 0.0148 |

Supplementary Table 6.
*Frontoparietal network attack tolerance difference summaries across costs*. Random differences are presented mean±SEM, based off 5000 iterations comparing attack tolerance in both groups across 36-node networks (i.e., identical in size to the frontoparietal network) excluding frontoparietal network nodes. P-value is a count of the number of times out of 5000 where the *random difference score > real difference score*.

| Cost | Attack Tolerance Difference | | P-value |
| --- | --- | --- | --- |
|  | Real | Random |  |
| 0.025 | 0.0131 | 6.76E-04±7.78E-05 | 0.0112 |
| 0.050 | 0.0471 | -3.95E-04±2.43E-04 | 0.0014 |
| 0.075 | 0.1022 | -0.0063±6.21E-04 | 0.0066 |
| 0.100 | 0.2101 | -0.0113±0.001 | 0.0026 |
| 0.125 | 0.2422 | -0.0112±0.0013 | 0.002 |
| 0.150 | 0.4153 | -0.0166±0.0013 | <0.001 |
| 0.175 | 0.4932 | -0.0177±0.0013 | <0.001 |
| 0.200 | 0.5370 | -0.0193±0.0012 | <0.001 |
| 0.225 | 0.5714 | -0.0195±0.0011 | <0.001 |
| 0.250 | 0.6345 | -0.0187±9.9E-04 | <0.001 |

Supplementary Table 7.

*Frontoparietal network,* ***excluding cerebellar ROIs****, targeted attack analysis summary statistics across costs.* Values presented are mean±SEM of area under the curve.

| Cost | Parkinson’s disease groups | | P-value |
| --- | --- | --- | --- |
|  | Cognitive Decline | Cognitive Stable |  |
| 0.025 | 0.0586±0.0038 | 0.0615±0.0040 | 0.6174 |
| 0.050 | 0.1800±0.0079 | 0.1914±0.0085 | 0.358 |
| 0.075 | 0.3928±0.0168 | 0.4472±0.0196 | 0.0468 |
| 0.100 | 0.6764±0.0258 | 0.7503±0.0336 | 0.085 |
| 0.125 | 1.005±0.0358 | 1.132±0.0356 | 0.0204 |
| 0.150 | 1.374±0.0527 | 1.523±0.0577 | 0.0784 |
| 0.175 | 1.759±0.0652 | 1.913±0.0649 | 0.127 |
| 0.200 | 2.111±0.0788 | 2.306±0.0754 | 0.0936 |
| 0.225 | 2.363±0.0856 | 2.663±0.0836 | 0.0272 |
| 0.250 | 2.710±0.0891 | 3.059±0.0907 | 0.0128 |

Supplementary Table 8.

*Auditory network targeted attack analysis summary statistics across costs.* Values presented are mean±SEM of area under the curve.

| Cost | Parkinson’s disease groups | | P-value |
| --- | --- | --- | --- |
|  | Cognitive Decline | Cognitive Stable |  |
| 0.025 | 0.0045±0.0006 | 0.0055±0.0011 | 0.4870 |
| 0.050 | 0.0158±0.0021 | 0.0165±0.0031 | 0.8766 |
| 0.075 | 0.0538±0.0054 | 0.0511±0.0055 | 0.7396 |
| 0.100 | 0.0988±0.0079 | 0.1043±0.0095 | 0.6620 |
| 0.125 | 0.1201±0.0077 | 0.1379±0.0102 | 0.1706 |
| 0.150 | 0.1955±0.0131 | 0.2280±0.0162 | 0.1276 |
| 0.175 | 0.2670±0.0150 | 0.3275±0.0219 | 0.0242 |
| 0.200 | 0.3097±0.0173 | 0.3795±0.0237 | 0.0172 |
| 0.225 | 0.4044±0.0201 | 0.4496±0.0252 | 0.1782 |
| 0.250 | 0.5018±0.0211 | 0.5358±0.0248 | 0.3154 |

Supplementary Table 9.

*Whole brain weighted degree summary statistics across costs.* Values presented are mean±SEM.

| Cost | Parkinson’s disease groups | | P-value |
| --- | --- | --- | --- |
|  | Cognitive Decline | Cognitive Stable |  |
| 0.000 | 0.0±0.0 | 0.0±0.0 | NA |
| 0.050 | 7.252±0.2555 | 7.431±0.2549 | 0.2112 |
| 0.100 | 11.73±0.3171 | 12.01±0.3199 | 0.2322 |
| 0.150 | 15.20±0.3440 | 15.56±0.3496 | 0.2488 |
| 0.200 | 17.99±0.3553 | 18.42±0.3620 | 0.2448 |
| 0.250 | 20.24±0.3584 | 20.73±0.3659 | 0.2342 |

Supplementary Table 10*.*

*Frontoparietal network weighted degree summary statistics across costs.* Values presented are means±SEM.

| Cost | Parkinson’s disease groups | | P-value |
| --- | --- | --- | --- |
|  | Cognitive Decline | Cognitive Stable |  |
| 0.000 | 0.0±0.0 | 0.0±0.0 | NA |
| 0.050 | 7.683±0.7248 | 7.519±0.6601 | 0.6488 |
| 0.100 | 12.30±0.8820 | 12.22±0.8336 | 0.8720 |
| 0.150 | 15.77±0.9448 | 15.73±0.9023 | 0.9380 |
| 0.200 | 18.53±0.9631 | 18.52±0.9348 | 0.9846 |
| 0.250 | 20.72±0.9638 | 20.79±0.9440 | 0.9016 |

Supplementary Table 11.

*Parkinson’s disease vs. control group comparisons of frontoparietal network attack tolerance.* Values presented are means±SEM of area under the curve.

| Cost | Groups | | P-value |
| --- | --- | --- | --- |
|  | Parkinson’s Disease | Controls |  |
| 0.025 | 0.0938±0.0034 | 0.0983±0.0057 | 0.5044 |
| 0.050 | 0.3228±0.0099 | 0.3239±0.0155 | 0.9570 |
| 0.075 | 0.6508±0.0197 | 0.7207±0.0395 | 0.0832 |
| 0.100 | 1.135±0.0353 | 1.389±0.0733 | 0.0006 |
| 0.125 | 1.670±0.0547 | 2.043±0.0962 | 0.0012 |
| 0.150 | 2.217±0.0721 | 2.675±0.1187 | 0.0014 |
| 0.175 | 2.722±0.0866 | 3.232±0.1165 | 0.0032 |
| 0.200 | 3.264±0.0935 | 3.746±0.1208 | 0.0068 |
| 0.225 | 3.787±0.0951 | 4.230±0.1242 | 0.0142 |
| 0.250 | 4.226±0.0970 | 4.621±0.1270 | 0.0300 |

Supplementary Table 12.

*Frontoparietal network targeted attack analysis summary statistics across costs, cognitively declining PD, cognitively stable PD, controls***.** Values presented are means±SEM.

| Cost | Parkinson’s disease groups | | Control group | P-value | Post-hoc p-values, FDR corrected | | |
| --- | --- | --- | --- | --- | --- | --- | --- |
|  | Decline | Stable |  |  | Decline vs. stable | Decline vs. control | Stable vs. control |
| 0.025 | 0.0890±0.0044 | 0.1020±0.0051 | 0.0983±0.0057 | p>0.05 | - | - | - |
| 0.050 | 0.3053±0.0129 | 0.3524±0.0135 | 0.3239±0.0155 | p>0.05 | - | - | - |
| 0.075 | 0.6127±0.0250 | 0.7149±0.0274 | 0.7207±0.0395 | 0.0144 | 0.0303 | 0.0303 | 0.9042 |
| 0.100 | 1.057±0.0434 | 1.267±0.0498 | 1.389±0.0733 | 0.0002 | 0.0072 | 0.0012 | 0.1688 |
| 0.125 | 1.580±0.0707 | 1.822±0.0775 | 2.043±0.0962 | 0.0002 | 0.0414 | 0.0012 | 0.0774 |
| 0.150 | 2.062±0.0872 | 2.477±0.1072 | 2.675±0.1187 | 0.0002 | 0.0078 | 0.0012 | 0.2142 |
| 0.175 | 2.538±0.1088 | 3.031±0.1189 | 3.232±0.1165 | 0.0002 | 0.0072 | 0.0006 | 0.2376 |
| 0.200 | 3.064±0.1190 | 3.601±0.1235 | 3.746±0.1208 | 0.0002 | 0.0072 | 0.0036 | 0.4172 |
| 0.225 | 3.574±0.1249 | 4.145±0.1104 | 4.230±0.1242 | 0.0008 | 0.0066 | 0.0036 | 0.6130 |
| 0.250 | 3.989±0.1283 | 4.624±0.1010 | 4.621±0.1270 | 0.0002 | 0.0036 | 0.0036 | 0.9854 |

Supplementary Table 13.

*Parkinson’s disease vs. control group comparisons of frontoparietal network weighted degree summary statistics across costs.* Values presented are means±SEM.

| Cost | Groups | | P-value |
| --- | --- | --- | --- |
|  | Parkinson’s Disease | Controls |  |
| 0.000 | 0.0±0.0 | 0.0±0.0 | NA |
| 0.050 | 7.622±0.7006 | 5.782±0.6593 | 0.0002 |
| 0.100 | 12.27±0.8640 | 9.663±0.8592 | 0.0002 |
| 0.150 | 15.75±0.9289 | 12.71±0.9630 | 0.0002 |
| 0.200 | 18.52±0.9526 | 15.20±1.033 | 0.0002 |
| 0.250 | 20.75±0.9564 | 17.26±1.079 | 0.0002 |

Supplementary Table 14.

*Frontoparietal network weighted degree summary statistics across costs, cognitively declining PD, cognitively stable PD, controls***.** Values presented are means±SEM.

| Cost | Parkinson’s disease groups | | Control group | P-value | Post-hoc p-values, FDR corrected | | |
| --- | --- | --- | --- | --- | --- | --- | --- |
|  | Decline | Stable |  |  | Decline vs. stable | Decline vs. control | Stable vs. control |
| 0.000 | 0.0±0.0 | 0.0±0.0 | 0.0±0.0 | NA | NA | NA | NA |
| 0.050 | 7.683±0.7248 | 7.519±0.6601 | 5.782±0.6593 | 0.0004 | 0.6488 | 0.0003 | 0.0003 |
| 0.100 | 12.30±0.8820 | 12.22±0.8336 | 9.663±0.8592 | 0.0004 | 0.8720 | 0.0003 | 0.0003 |
| 0.150 | 15.77±0.9448 | 15.73±0.9023 | 12.71±0.9630 | 0.0004 | 0.9380 | 0.0003 | 0.0003 |
| 0.200 | 18.53±0.9631 | 18.52±0.9348 | 15.20±1.033 | 0.0004 | 0.9846 | 0.0003 | 0.0003 |
| 0.250 | 20.72±0.9638 | 20.79±0.9440 | 17.26±1.079 | 0.0004 | 0.9016 | 0.0003 | 0.0003 |

Supplementary Table 15.

*Frontoparietal network Montreal Neurological Institute (MNI) coordinates.* Asterisks indicate cerebellar nodes.

| Network Assignment | X | Y | Z |
| --- | --- | --- | --- |
| Frontoparietal | -52.6 | -48.83 | 42.5 |
| Frontoparietal | -46.5 | 10.85 | 23.04 |
| Frontoparietal | -43.93 | 1.8 | 45.7 |
| Frontoparietal | -42.23 | 38.21 | 21.35 |
| Frontoparietal | -42.09 | -54.98 | 44.74 |
| Frontoparietal | -42.1 | 24.68 | 29.53 |
| Frontoparietal | -41.06 | 5.81 | 32.72 |
| Frontoparietal | -41.68 | 45.16 | -2.31 |
| Frontoparietal | -34.16 | 54.83 | 4.36 |
| Frontoparietal | -28.4 | -57.93 | 47.78 |
| Frontoparietal | -22.53 | 10.76 | 63.73 |
| Frontoparietal | -2.98 | 26.41 | 44.42 |
| Frontoparietal | 24.07 | 44.61 | -15.35 |
| Frontoparietal | 31.83 | 14.37 | 55.98 |
| Frontoparietal | 33.38 | -53.12 | 44.02 |
| Frontoparietal | 33.6 | 54.22 | -12.95 |
| Frontoparietal | 37.45 | -64.7 | 40.38 |
| Frontoparietal | 38.37 | 43.18 | 15.06 |
| Frontoparietal | 39.87 | 18.39 | 39.72 |
| Frontoparietal | 43.93 | -52.95 | 46.95 |
| Frontoparietal | 43.25 | 49.25 | -2.31 |
| Frontoparietal | 47.01 | 9.93 | 32.66 |
| Frontoparietal | 47.98 | 24.56 | 26.5 |
| Frontoparietal | 49.18 | -42.41 | 45.16 |
| Frontoparietal | 58.31 | -52.79 | -13.61 |
| Frontoparietal | 47.6 | 22.16 | 9.74 |
| Frontoparietal | 55.27 | -44.59 | 36.7 |
| Frontoparietal | 14.15 | -1.19 | 18.18 |
| Frontoparietal | -15.19 | -1.5 | 18.84 |
| Frontoparietal* | -10 | -78 | -28 |
| Frontoparietal* | 10 | -78 | -28 |
| Frontoparietal* | -34 | -72.01 | -48 |
| Frontoparietal* | 34 | -72 | -48 |
| Frontoparietal* | -30.5 | -66 | -30 |
| Frontoparietal* | 31.68 | -62.83 | -30.4 |
| Frontoparietal* | 40 | -44 | -38 |

Supplementary Table 16.

*Auditory network Montreal Neurological Institute (MNI) coordinates.*

| Network | X | Y | Z |
| --- | --- | --- | --- |
| Auditory | -52.92 | -21.83 | 22.97 |
| Auditory | 55.96 | -5.03 | 13.25 |
| Auditory | 57.88 | -15.62 | 7.49 |
| Auditory | 65.43 | -33.2 | 19.97 |
| Auditory | 43.45 | -22.93 | 19.85 |
| Auditory | 59.4 | -17.34 | 28.69 |
| Auditory | -60.48 | -25.22 | 13.82 |
| Auditory | -55.22 | -9.42 | 11.73 |
| Auditory | -49.77 | -34.36 | 25.74 |
| Auditory | -49.14 | -26.3 | 5.18 |
| Auditory | -38.43 | -33.34 | 16.98 |
| Auditory | 31.75 | -26.33 | 12.91 |
