# Supplementary material for: Frontoparietal network resilience is associated with protection against cognitive decline in Parkinson’s disease
Source: Commun Biol. 2021 Sep 1;4:1021. doi: 10.1038/s42003-021-02478-3 (PMC8410800; doi:10.1038/s42003-021-02478-3)
Supplement: Supplementary file 2 — Reporting Summary [file 42003_2021_2478_MOESM2_ESM.pdf]

nature research

Corresponding author(s): Eran Dayan
Last updated by author(s): Jun 9, 2021

Reporting Summary

Nature Research wishes to improve the reproducibility of the work that we publish. This form provides structure for consistency and transparency in reporting. For further information on Nature Research policies, see our Editorial Policies and the Editorial Policy Checklist.

Statistics

For all statistical analyses, confirm that the following items are present in the figure legend, table legend, main text, or Methods section.

n/a	Confirmed	
☐	☒	The exact sample size (*n*) for each experimental group/condition, given as a discrete number and unit of measurement
☐	☒	A statement on whether measurements were taken from distinct samples or whether the same sample was measured repeatedly
☐	☒	The statistical test(s) used AND whether they are one- or two-sided<br>*Only common tests should be described solely by name; describe more complex techniques in the Methods section.*
☐	☒	A description of all covariates tested
☒	☐	A description of any assumptions or corrections, such as tests of normality and adjustment for multiple comparisons
☐	☒	A full description of the statistical parameters including central tendency (e.g. means) or other basic estimates (e.g. regression coefficient) AND variation (e.g. standard deviation) or associated estimates of uncertainty (e.g. confidence intervals)
☐	☒	For null hypothesis testing, the test statistic (e.g. *F*, *t*, *r*) with confidence intervals, effect sizes, degrees of freedom and *P* value noted<br>*Give P values as exact values whenever suitable.*
☒	☐	For Bayesian analysis, information on the choice of priors and Markov chain Monte Carlo settings
☒	☐	For hierarchical and complex designs, identification of the appropriate level for tests and full reporting of outcomes
☒	☐	Estimates of effect sizes (e.g. Cohen's *d*, Pearson's *r*), indicating how they were calculated

Our web collection on statistics for biologists contains articles on many of the points above.

Software and code

Policy information about availability of computer code

Data collection	No software was used for collection of data
Data analysis	MatLab R2019b<br>R version 4.0.2

For manuscripts utilizing custom algorithms or software that are central to the research but not yet described in published literature, software must be made available to editors and reviewers. We strongly encourage code deposition in a community repository (e.g. GitHub). See the Nature Research guidelines for submitting code & software for further information.

Data

Policy information about availability of data

All manuscripts must include a data availability statement. This statement should provide the following information, where applicable:

- Accession codes, unique identifiers, or web links for publicly available datasets
- A list of figures that have associated raw data
- A description of any restrictions on data availability

All data in the present study were extracted from the Parkinson’s Progression Markers Initiative (PPMI) database (http://www.ppmi-info.org).

Field-specific reporting

Life sciences study design

All studies must disclose on these points even when the disclosure is negative.

Sample size	No sample size calculation was performed. We used all data meeting the inclusion criteria from the Parkinson's Progression Markers Initiative database.
Data exclusions	Data were excluded according to study design constraints, including the availability of complete anatomical and resting-state functional MRI scans, and longitudinal global cognition data. Other exclusion criteria were as per PPMI's study protocol (https://www.ppmi-info.org/tag/study-protocol/)
Replication	N/A
Randomization	N/A
Blinding	N/A

Reporting for specific materials, systems and methods

We require information from authors about some types of materials, experimental systems and methods used in many studies. Here, indicate whether each material, system or method listed is relevant to your study. If you are not sure if a list item applies to your research, read the appropriate section before selecting a response.

Materials & experimental systems

n/a	Involved in the study
☒	☐ Antibodies
☒	☐ Eukaryotic cell lines
☒	☐ Palaeontology and archaeology
☒	☐ Animals and other organisms
☐	☒ Human research participants
☐	☒ Clinical data
☒	☐ Dual use research of concern

Methods

n/a	Involved in the study
☒	☐ ChIP-seq
☒	☐ Flow cytometry
☐	☒ MRI-based neuroimaging

Human research participants

Policy information about studies involving human research participants

Population characteristics	This information can be found at: https://www.ppmi-info.org/access-data-specimens/data-faq/
Recruitment	See above
Ethics oversight	All participants provided written informed consent, and the procedures were all in accordance with the approved regulations and guidelines of the Institutional Review Boards of participating study centers

Note that full information on the approval of the study protocol must also be provided in the manuscript.

Clinical data

Policy information about clinical studies

All manuscripts should comply with the ICMJEguidelines for publication of clinical research and a completedCONSORT checklist must be included with all submissions.

Clinical trial registration	NCT01141023
Study protocol	Study protocol can be found at: https://www.ppmi-info.org/study-design/
Data collection	https://www.ppmi-info.org/access-data-specimens/
Outcomes	https://www.ppmi-info.org/about-ppmi/study-goals/

Magnetic resonance imaging

Experimental design

Design type	Resting-state fMRI study

Design specifications	One resting-state (task-free) imaging session per subject
Behavioral performance measures	Montreal Cognitive Assessment (MoCA) scores

Acquisition

Imaging type(s)	Structural and functional
Field strength	3T
Sequence & imaging parameters	Anatomical scans were acquired using a magnetization prepared rapid acquisition gradient echo (MPRAGE) sequence with generalized autocalibrating partial parallel acquisition (GRAPPA) (TE = 2.98 ms, TR = 2300 ms, flip angle = 9˚, voxel size = 1 mm3). Resting-state fMRI scans were acquired using an echo-planar sequence (TE = 25 ms, TR = 2400 ms, 210 volumes, flip angle = 80˚, voxel size = 3.3 mm3). The resting-state scans were 8.4 minutes in length.
Area of acquisition	Whole brain

Diffusion MRI ☐ Used ☒ Not used

Preprocessing

Preprocessing software	Preprocessing of imaging data was performed via MATLAB R2019b (MathWorks, Natick, MA, USA), using SPM 12 and the CONN toolbox version 18.b. Complete documentation of the following CONN pipelines can be found online (https://web.conn-toolbox.org/fmri-methods).<br>CONN's default preprocessing pipeline for volume-based analyses was used. During preprocessing, functional images were realigned, unwarped, and slice-time corrected. Outlier volumes were identified and scrubbed, using the intermediate settings of 0.9mm subject motion as measured by framewise displacement and a global signal threshold of Z = 5. Functional data was normalized into standard Montreal Neurological Institute (MNI) space and grey-matter, white-matter, and cerebrospinal fluid were segmented. Finally, functional data was spatially smoothed with a Gaussian kernel of 8mm full-width half maximum.
Normalization	Direct normalization to MNI-space
Normalization template	MNI152
Noise and artifact removal	CONN's default denoising pipeline was used. White matter, cerebrospinal fluid, and 12 subject-motion parameters were included as regressors in the denoising step. A temporal band-pass filter was also applied to remove temporal frequencies below 0.008 Hz or above 0.09 Hz from the blood-oxygen level dependent (BOLD) signal.
Volume censoring	Subjects (n=1) with >50% of volumes removed were excluded from analyses.

Statistical modeling & inference

Model type and settings	Group comparisons were evaluated using a non-parametric pair-wise permutation test. The aovperm function from the permuco package in R was used. Five thousand permutations were performed for each test, and significance level was set at p=0.05.
Effect(s) tested	Pairwise group comparisons of network resilience (area under the curve of global efficiency vs. cost plots) and weighted degree.

Specify type of analysis: ☐ Whole brain ☒ ROI-based ☐ Both

Anatomical location(s)	300 functionally defined spherical cortical-subcortical-cerebellar ROIs (as defined by Seitzman et al., 2020)
Statistic type for inference (See Eklund et al. 2016)	Not applicable.
Correction	Not applicable. Comparisons were pairwise.

Models & analysis

n/a	Involved in the study
☐	☒ Functional and/or effective connectivity
☐	☒ Graph analysis
☒	☐ Multivariate modeling or predictive analysis

Functional and/or effective connectivity	Fisher-Z transformed bivariate correlations
Graph analysis	Whole-brain and single-network targeted attack analyses were performed on proportionally thresholded matrices (2.5-25%). Nodes were iteratively removed in descending order of degree. Global efficiency was calculated after each attack iteration, yielding an overall curve denoting values of global efficiency as a function of fraction of nodes removed.
